# Inflammatory Proteins and Clinical Response to Psychological Therapy in Patients with Depression: An Exploratory Study

**DOI:** 10.3390/jcm9123918

**Published:** 2020-12-02

**Authors:** Rebecca Strawbridge, Lindsey Marwood, Sinead King, Allan H. Young, Carmine M. Pariante, Alessandro Colasanti, Anthony J. Cleare

**Affiliations:** 1 Department of Psychological Medicine, Institute of Psychiatry, Psychology & Neuroscience, King’s College London, Denmark Hill, London SE5 8AZ, UK; lindsey.marwood@kcl.ac.uk (L.M.); sinead.king@kcl.ac.uk (S.K.); allan.young@kcl.ac.uk (A.H.Y.); carmine.pariante@kcl.ac.uk (C.M.P.); anthony.cleare@kcl.ac.uk (A.J.C.); 2 South London & Maudsley NHS Foundation Trust, Denmark Hill, London SE5 8AZ, UK; 3 Department of Neuroscience, Brighton and Sussex Medical School, Sussex University, Brighton BN1 9PX, UK; a.colasanti@bsms.ac.uk

**Keywords:** inflammation, depression, psychological therapy, treatment response, cytokines

## Abstract

In people with depression, immune dysfunctions have been linked with treatment non-response, but examinations of psychological therapy outcomes, particularly longitudinal biomarker studies, are rare. This study investigated relationships between inflammation, depressive subtypes and clinical outcomes to psychological therapy. Adults with depression (*n* = 96) were assessed before and after a course of naturalistically-delivered psychological therapy. In total, 32 serum inflammatory proteins were examined alongside therapy outcomes and depressive subtypes (somatic/cognitive symptom subtype, and bipolar/unipolar depression). Overall, 49% of participants responded to treatment. High levels of tumour necrosis factor (TNFα), interleukin-6 (IL-6) and soluble intracellular adhesion molecule-1 (sICAM1), and low interferon-γ (IFNγ), preceded a poorer response to therapy. After therapy, non-responders had elevated c-reactive protein (CRP), thymus and activation-regulated chemokine (TARC) and macrophage chemoattractant protein-4 (MCP4), and attenuated IFNy. Non-somatic depressive symptoms were universally not associated with proteins, while somatic-depressive symptom severity was positively correlated with several pro-inflammatory markers. In the somatic subgroup only, IL-6 and serum amyloid alpha (SAA) decreased between pre- and post-therapy timepoints. Regardless of treatment response, IL-7, IL-8, IL-15 and IL-17 increased over time. These results suggest that inflammation is associated with somatic symptoms of depression and non-response to psychological therapy. Future work may enhance the prospective prediction of treatment-response by examining larger samples of individuals undertaking standardised treatment programmes.

## 1. Introduction

Since the macrophage hypothesis of depression was proposed three decades ago, associations between mood disorders and several aspects of the immune system have been examined in a plethora of studies. Many early studies focused on innate immune cells and macrophages; inflammasome protein signalling cascades in pro-inflammatory and regulatory inflammatory responses have identified some key proteomic biomarkers of mood disorders (that may be trans-diagnostically important across depressive and anxiety disorders), particularly pro-inflammatory cytokines and acute phase proteins [1].

Increasing evidence suggests that dysregulation of the inflammatory system plays a role in clinical responses to treatments for people with depressive disorders [2,3]. Several studies have indicated that elevated levels of pro-inflammatory cytokines (messenger proteins of the immune system) precede a poorer outcome to conventional antidepressants [4]. While psychotropic medications appear to affect inflammatory activity, these effects vary between agents and across studies [1,5] making it challenging to disentangle the effects of pharmacological treatment and clinical improvements in understanding longitudinal inflammatory alterations in depression.

The existence of non-biological interventions for mood disorders can facilitate an observation of inflammatory changes during treatment that are not purely attributable to biological treatment effects. However, this opportunity has rarely been taken advantage of in assessing response to treatments. Two recent meta-analyses have assessed effects of psychological therapies on immune activity from randomised controlled trials (RCTs), indicating a downregulatory effect of therapy on inflammation that may be driven by c-reactive protein (CRP) [6] and may not be as reliable an effect for major depressive disorder (MDD) (5 studies; ES = 0.28 [95%CI −0.19 to 0.75]) as for some inflammatory conditions (overall effect across all illness types (56 studies); ES = 0.30 [95%CI 0.21 to 0.40]) [7]. A systematic review examining only cognitive behavioural therapy (CBT) effects on inflammation in those with depression (across different study designs) indicated some anti-inflammatory effects of CBT in 14/23 studies, an association of inflammatory changes to symptom alterations in 2/3 studies and a predictive effect of pre-treatment inflammation on clinical outcomes in 3/3 studies, suggesting that those with elevated inflammation respond less well to CBT [8]. A more recent systematic review [9] examined 12 studies (11 of depression comorbid with other conditions) of inflammatory associations with psychotherapies in RCTs; no significant effects of therapy on inflammation were identified and only one study [10] had assessed inflammatory predictors of response (finding elevated CRP preceding a poorer response to therapies, not assessing cytokines longitudinally) [9]. 

One likely reason for inconsistent findings across studies in this literature pertains to heterogeneity between patients; abnormal inflammation appears clearly in a subset, but not in the whole population of those suffering with depression [11,12]. Most evidence of subgroups in depression has focused on atypical and melancholic subtypes; most frequently atypical depression has been associated with aberrant inflammation [11], but studies have also reported comparably elevated inflammation in melancholic and atypical [13] or non-melancholic depression [14]. A wide array of other depressive symptoms that are not accounted for by currently-defined subtypes have also been associated with increased inflammation, e.g., those categorised as neurovegetative [15], somatic [16,17] or reflective of sickness behaviour [18]. Consensus has not been reached on the precise symptoms a subtype such as this would include but its conceptualisation could incorporate biological (such as an aberrant inflammatory profile) as well as clinical features. People with bipolar depression more often present with an atypical subtype and may have a higher propensity for dysregulated inflammation (related to, for example, lifestyle, medication or physical health factors), although bidirectional and inconsistent findings have previously been reported between the few studies that have directly compared patients with unipolar versus bipolar depression [19,20,21]. Studies have seldom grouped participants according to ‘somatic’ or ‘cognitive’ (or similar) subtypes alongside inflammatory markers and these require longitudinal assessment in association with treatment outcomes. If a somatic subgroup is reliably demonstrated to be distinct from other depressed groups in terms of inflammation, it may provide indications for treatment stratification to improve clinical outcomes.

### Study Objectives

This exploratory study aimed to improve understanding of how depression subtyping could affect the relationship between a large panel of serum inflammatory proteins, assessed longitudinally, and response to psychological treatment. Specifically, based on the accumulated literature, the following null hypotheses were proposed for measurements before treatment, after treatment, and in changes between time points:

(1) Levels of inflammatory proteins will not differ between depressed patients who do, and do not, respond to psychological treatment, either prior to treatment (predictors of response) or afterwards (cross-sectional associations)
(2) Inflammatory protein levels will not change between pre- and post-treatment measurements;
(3) Participants categorised as having distinct subtypes of depression (i.e., somatic versus cognitive; bipolar versus unipolar) will not differ from other participants in terms of inflammation or the association between inflammation and treatment response.

It was anticipated that our null hypotheses will be rejected for some, but not all, biomarkers. The a priori markers expected to demonstrate significant associations with treatment-response are CRP, tumour necrosis factor (TNFα), and interleukin-6 (IL-6), based on previous findings [21].

## 2. Methods

### 2.1. Design

This study was a longitudinal, naturalistic treatment investigation of individuals with depression who undertook psychological therapy in an Improving Access to Psychological Therapy (IAPT) NHS service in London, UK. Prior to recruitment, ethical approval was obtained from Bromley NHS Research Ethics Committee (references 13/LO/1347, 13/LO/1897). Participants were assessed before treatment (after referral and IAPT assessment but before starting therapy) and after treatment (2–6 weeks following intervention completion without plans to start new therapy).

### 2.2. Participants

Participants were identified following referral for IAPT psychological therapies. A subsample of the ‘PROMPT’ study (see [22,23] for more details), the majority of participants (*n* = 87/96) were contacted by the PROMPT team after consenting via IAPT to be contacted by researchers; the other individuals made contact with the study team in response to community advertisements. The two subsamples were comparable in terms of data collection and intervention methods. Participants provided fully informed consent after receiving written and verbal study information and prior to commencing the baseline research assessment.

Participants were included in this study if their scores exceeded standardised cut-offs (≥10) on the Patient Health Questionnaire (PHQ9) [24], indicating current depression, and if they had been referred for an IAPT course of psychological therapy. While initially 99 participants were included, 3 did not undertake any treatment (no sessions attended *n* = 2, referred to non-psychological service *n* = 1), leaving a final sample of 96 participants who had completed therapy. Treatment completion was defined as attendance at ≥4 therapy sessions. All participants completed clinical outcome data before and after therapy. Inflammatory data were present for all participants before therapy but after treatment was only collected from 50 participants (17 were uncontactable, 28 unavailable to attend blood collection within study timeframes, 1 needle phobia).

### 2.3. Measures

#### 2.3.1. Treatment-Outcome Measures

The PHQ9 [24] was used to assess the severity of depressive symptoms at both pre- and post- treatment measurements. Individuals with a total score reduction of ≥50% between pre- and post-treatment (or a score of <5 after treatment, indicating remission) [24] were categorised as treatment responders, and others as non-responders.

#### 2.3.2. Depression-Subtype Measures

Using the methods reported by Duivis et al. [17] symptom domains were categorised as ‘somatic’ (PHQ9 items pertaining to fatigue and disturbances to sleep, appetite and psychomotor function) or ‘cognitive’ (comprising PHQ9 items representing the more psychological symptoms of depressed mood, anhedonia, guilt, concentration difficulties and suicidal ideation). Severity scores for both ‘somatic’ and ‘cognitive’ subscales were calculated by totalling item scores. Participants with a higher somatic than cognitive score were considered as having somatic-type depression; a greater proportion of the total PHQ9 score is comprised of cognitive symptoms and participants with equal scores for each were categorised as somatic for this reason. 

The Mini International Psychiatric Interview (MINI) [25] was conducted at baseline from which presence or absence of a bipolar disorder was calculated (i.e., by meeting criteria for a history of hypomania or mania). Five participants had not completed the MINI and to avoid the need for missing data, scores from the self-rated Hypomanic Checklist (HCL-16) was instead used to categorise participants as having bipolar or unipolar depression (using a cut-off score of ≥8) [26]. 

#### 2.3.3. Additional Exploratory Outcome Measures

The remaining measures were completed at the pre-treatment assessment only. Age, gender and BMI were measured as the most likely covariates. Other putative covariates included physical illness (assessed using the Modified Cumulative Illness Rating Scale (MCIRS) [27] with the total score excluding the mental health item), psychotropic medication use, early life stress examined using the Childhood Trauma Questionnaire (CTQ) [28] and number of current comorbidities as assessed by the MINI [25]. Due to the small sample size, attempts were made to limit the number of confounding factors examined and maximise statistical power; thus, the number of comorbidities excluded bipolarity (assessed separately) but included all other current diagnoses on the MINI, i.e., panic disorder, agoraphobia, social phobia, obsessive compulsive disorder, post-traumatic stress disorder, alcohol or other drug dependence or abuse, psychotic disorders (including psychotic depression), anorexia, bulimia, generalised anxiety disorder and antisocial personality disorder. A further factor that has been implicated in inflammatory depression is treatment-resistance [21] assessed within the current episode of depression, using the Maudsley Staging Method (MSM) [29].

#### 2.3.4. Inflammatory Measures

Blood was collected (1 × 5 mL tube) and allowed to clot before centrifugation and extraction of serum, which was transferred into cryovials and frozen at ×80 °C. Serum concentrations of biomarkers were assayed using a high-sensitivity Meso Scale Discovery V-Plex kit (Meso Scale Diagnostics, Maryland, USA). The panel of biomarkers comprised the following proteins: CRP, IFNγ, IL-10, IL-12, IL-12p70, IL-13, IL-15, IL-16, IL-17, IL-1α, IL-1β, IL-2, IL-4, IL-5, IL-6, IL-7, IL-8 (CXCL8), TNFα, TNFβ, Eotaxin (CCL11), Eotaxin-3 (CCL26), GM-CSF, IP-10 (CXCL10), MCP1 (CCL2), MCP4 (CCL13), Mip1a (CCL3), Mip1b (CCL4), serum amyloid A (SAA), sICAM1 (sCD54), sVCAM1 (sCD106), TARC (CCL17) and Tie2. Unless reported otherwise, biomarker levels are expressed as picograms per millilitre (pg/mL).

### 2.4. Data Analysis

Inflammatory markers and clinical variables were measured before and after psychological therapy.

#### 2.4.1. Data Cleaning

Coefficients of determination (r^2^) of standard curves were calculated and raw data examined. Proteins with ≥50% values not detected by the assay at either time point were excluded. Non-detected biomarker data were imputed using LLOD/2 [30] and all markers were transformed (log base 10) prior to parametric test analysis. Normality of data distribution was assessed for all variables by examining skewness and kurtosis values, frequency distribution (histogram plots), stem and leaf plots, P-P (probability-probability) plots, Q-Q (quantile-quantile) plots and box plots, and the Kolmogorov–Smirnov test. Analyses included a bootstrap of 1000 resamples. 

#### 2.4.2. Main Comparisons

The main comparisons focused on inflammatory associations with treatment outcomes and over time, in accordance with hypotheses. For each marker, the following comparisons were undertaken:

(a) Logistic regressions of pre-treatment protein levels (independent variables) as potential predictors of subsequent response to psychological therapy (dependent variable), adjusting for age, gender and BMI (covariates). Using the same approach, post-treatment protein markers were compared between responders and non-responders.
(b) 2 × 2 mixed-factorial ANOVA analyses to assess potential inflammatory changes occurring between pre- and post-treatment assessments (within-subjects) and between responders and non-responders (time by group interaction).
(c) Analysis of inflammatory associations between participants with a predominantly somatic vs. cognitive depression subtype: between-subjects cross-sectional associations through independent-samples t-tests at each timepoint and protein changes over time between these subgroups assessed using a 2 × 2 mixed-factorial ANOVA test.
(d) Compare bipolar and unipolar depressed participants, using the same methods as above.

We elected, a priori, to report and focus on uncorrected *p* values < 0.05 as nominally significant. This decision was taken since this is essentially an exploratory study, with relatively low statistical power and in this context, use of ‘correction’ for multiple testing is relatively conservative, leading to the possibility of type II errors which would prevent the detection of potentially important signals, as previously reported [31]. However, all of the main analyses were subsequently subjected to a Simes control for multiple testing (reported in text and tables) to provide indications of where a type I error may have occurred [32]. 

Secondary analyses were undertaken to:

(e) Determine whether inflammatory changes were associated with concomitant psychotropic medication use, using the methods outlined in comparison (c) to compare somatic and cognitive subtypes of depression.
(f) Assess inflammatory marker associations with treatment resistance (MSM score), using Pearson’s correlations.
(g) Assess inflammatory associations with overall severity, somatic and cognitive symptom severity subscales separately, using the same methods as above.

Additionally, correlations compared inflammatory protein levels with additional continuous baseline factors (age, BMI, physical illness severity, number of psychiatric comorbidities, early-life stress) while independent samples t-tests compared inflammation between dichotomous groups (gender, psychotropic medication status).

## 3. Results

### 3.1. Sample Characteristics

Participants had moderately severe depression at baseline (according to PHQ9 score categories; mean score = 18.4, standard deviation [SD] = 4.4). All 96 participants undertook psychological treatment (averaging 12 sessions over 7 months), of which 47 participants (49%) met criteria for treatment-response and 49 (51%) were non-responders. See Table 1 for characteristics of the responder and non-responder subsamples. Non-responders had more severe depression and anxiety symptoms at pre- as well as post-treatment timepoints and had more psychiatric comorbidities. 

### 3.2. Treatment Characteristics

In total, 75% of the sample were taking at least one medication, none of which were non-steroidal anti-inflammatory drugs (NSAIDs). In total, 47% were taking antidepressant medication (38% of subsequent responders, 55% non-responders). The therapies received by participants reflected current NHS provision within IAPT services; thus, 57 subjects had undertaken CBT-based therapy, 26 had a counselling-based approach, 3 had a short-term dynamic-focused psychotherapy and 10 participants had more than one type. There were more non-responders in the latter group, which likely reflects more extensive treatment provision to treatment-resistant patients. All participants attended >4 therapy sessions and the majority attended ≥6 sessions (only 4 participants had <6 sessions; 1 responder, 3 non-responders). 

### 3.3. Inflammatory Marker Characteristics

All proteins appeared accurately determined (all standard curves with r^2^ > 0.99). Of the 32 proteins, TNFβ and Eotaxin-3 had <10% undetected levels which were subjected to imputation and should be interpreted with due caution. Nine biomarkers all had ≥50% values not detected (IL-12p70, IL-13, GM-CSF, IL-4, IL-2, IL-1α, IL-1β, Mip1a and IL-5) and were excluded from analyses. Thus, 23 proteins were examined (see Supplementary Materials S1 for mean values). Following transformation, all proteins met criteria for normal distribution and all continuous non-biological variables were also normally distributed. 

### 3.4. Main Comparisons

We note that the majority of findings did not survive Simes adjustment for multiple testing (see below; paragraph prior to secondary comparisons, as well as Table 2 and Table 3).

#### 3.4.1. Inflammatory Predictors of Response

At pre-treatment, elevated IL-6 (OR = 0.154 [95% CI −4.179, −0.117], *p* = 0.035), TNFα (OR = 0.006 [95% CI −10.951, −0.645], *p* = 0.026) and sICAM1 (OR = 0.013 [95% CI −9.708, −0.073], *p* = 0.034), and attenuated IFNγ (OR = 5.664 [95% CI 0.195, 4.411], *p* = 0.049) predicted non-response to therapy (see Table 2).

After treatment, non-responders had higher CRP (OR = 0.234 [95% CI −3.659, −0.015], *p* = 0.023), MCP4 (OR = 0.014 [95% CI −12.775, −0.051], *p* = 0.038) and TARC (OR = 0.039 [95% CI −7.459, −1.344], *p* = 0.001) and lower IFNγ (OR = 9.331 [95% CI 0.294, 5.996], *p* = 0.026). When not adjusting for age, gender and BMI, both IL-6 (OR = 0.062, *p* = 0.027) and sICAM1 (OR = 0.002, *p* = 0.037) were also elevated significantly in non-responders; see Table 2. See Supplementary Materials S2 for post-hoc analyses of inflammatory associations with response (prospective and cross-sectional) adjusting for additional confounders.

#### 3.4.2. Longitudinal Inflammatory Changes with Treatment

**Figure 1:**
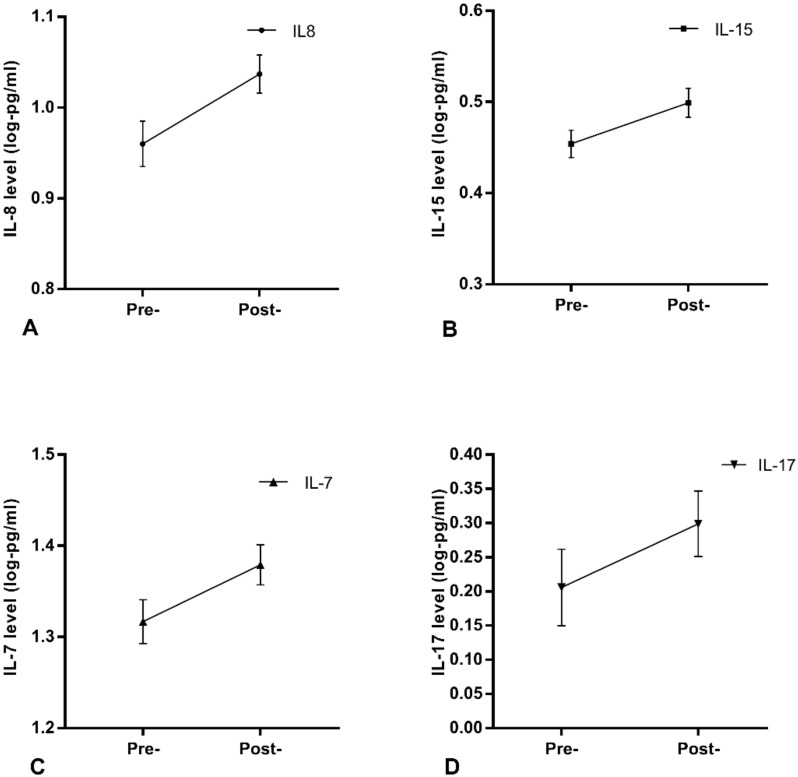
Protein increases with psychological therapy.

Between pre- and post-treatment assessments, an increase was observed in levels of IL-8 (F(1,48) = 14.600, *p* < 0.001), IL-15 (F(1,48) = 9.826, *p* = 0.003), IL-7 (F(1,48) = 7.181, *p* = 0.010) and IL-17 (F(1,48) = 5.049, *p* = 0.027). These changes are depicted in Figure 1 and all longitudinal results in Table 3.

Log transformed mean values and error bars (SEM) are presented. Note that axes have been cut according to protein levels expressed to depict differences clearly. For all proteins, *n* = 50. Log pg/mL as follows:

*A:* IL-8: Pre-treatment mean = 0.96, SEM = 0.025, Post-treatment mean = 1.037, SEM = 0.021.
*B:* IL-15: Pre-treatment mean = 0.454, SEM = 0.015, Post-treatment mean = 0.499, SEM = 0.016.
*C:* IL-7: Pre-treatment mean = 1.317, SEM = 0.024, Post-treatment mean = 1.379, SEM = 0.022.
*D:* IL-17: Pre-treatment mean = 0.206, SEM = 0.056, Post-treatment mean = 0.299, SEM = 0.048.

#### 3.4.3. Subgrouping Effects on Inflammatory Associations

Bipolar and unipolar depression subtype: In cross-sectional comparisons, no significant differences in inflammatory markers were identified between participants with unipolar and bipolar depression. Two pro-inflammatory markers showed increases between timepoints in participants with unipolar depression but decreases in those with bipolar depression: time by group interactions sVCAM1 F(1,48) = 5.006, *p* = 0.030 and Tie2 F(1,48) = 5.089, *p* = 0.029.

**Figure 2:**
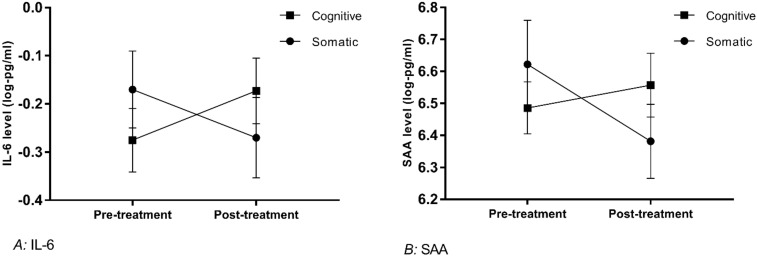
IL-6 and serum amyloid alpha (SAA) changes with therapy in somatic vs. cognitive symptom subtype.

Cognitive versus somatic subtype (grouping): No association between subtype and response to therapy was identified (X^2^ = 0.231, *p* = 0.810). Participants categorised as having a predominantly somatic subtype had higher IL-7 at baseline (t(76) = 2.38, *p* = 0.021). This group of participants showed decreases in SAA during treatment in contrast with small increases in the cognitive subgroup (time x group interaction F(1,38) = 4.881, *p* = 0.033). The same pattern was observed for IL-6 (interaction F(1,38) = 6.329, *p* = 0.016), both depicted in Figure 2.

Log transformed mean values over time (error bars show SEM); interaction with symptom subtype. Note that axes have been cut according to protein levels expressed, to depict differences clearly.

*A:* IL-6: For clarity of the Figure 2, a value of 1 has been added to protein data in the graph. Log values for participants with a somatic subtype (*n* = 16): pre-treatment mean = −0.17, SEM = 0.08, post-treatment mean = −0.27, SEM = 0.083. Participants with a cognitive subtype (*n* = 24): pre-treatment mean = −0.275, SEM = 0.066, post-treatment mean = −0.173, SEM = 0.068.
*B:* SAA: Log values for participants with a somatic subtype (*n* = 16): pre-treatment mean = 6.62, SEM = 0.14, post-treatment mean = 6.38, SEM = 0.12. Participants with a cognitive subtype (*n* = 24): pre-treatment mean = 6.49, SEM = 0.08, post-treatment mean = 6.56, SEM = 0.10.

#### 3.4.4. Correction for Multiple Comparisons

The majority of comparisons above did not survive Simes control for multiple comparisons. The exceptions were increases in IL-8 and IL-15 between pre- and post-treatment assessments. 

### 3.5. Secondary Comparisons

#### 3.5.1. Cross-Sectional Associations between Inflammatory Markers and Depression Severity

At baseline, three pro-inflammatory markers’ levels were positively associated with overall depression severity (PHQ total score) and the somatic subscale score: TNFa (r = 0.275, *p* = 0.007 and r = 0.354, *p* = 0.001 respectively), Mip1b (r = 0.250, *p* = 0.014 and r = 0.288, *p* = 0.011) and sICAM1 (r = 0.276, *p* = 0.007 and r = 0.268, *p* = 0.018). IL-6 was only (positively) associated with the somatic subscale severity (r = 0.252, *p* = 0.027), while IL-7 was only (inversely) associated with overall severity (r = −0.208, *p* = 0.042) and neither somatic nor cognitive subscales.

After treatment, two inflammatory markers were associated with both overall severity and the somatic subscale: IFNy, inversely correlated (overall severity r = −0.327, *p* = 0.020; somatic severity r = −0.325, *p* = 0.024) and TARC, positively correlated (overall severity r = 0.346, *p* = 0.014 and somatic severity r = 0.385, *p* = 0.007). Four additional markers were only (and positively) associated with the somatic subscale severity: MCP1 (r = 0.291, *p* = 0.045), MCP4 (r = 0.300, *p* = 0.038), sICAM1 (r = 0.315, *p* = 0.029) and SAA (r = 0.313, *p* = 0.030). No proteins were associated with the cognitive subscale score at either time point. 

#### 3.5.2. Influences of Concomitant Medication on Inflammation 

**Figure 3:**
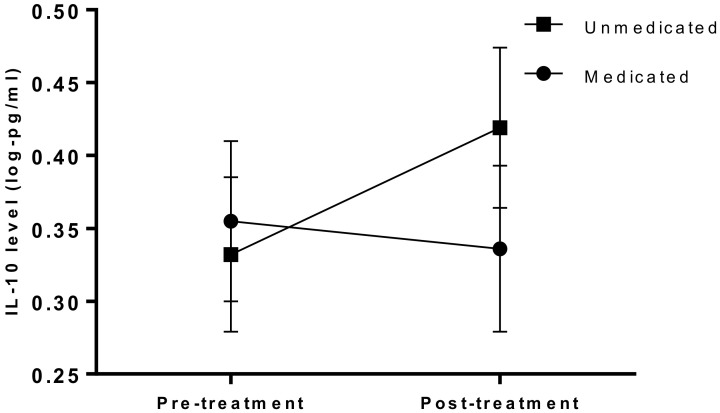
IL-10 changes with treatment; interaction with psychotropic medication.

Participants taking psychotropic medication at baseline had lower IL-17 (t(94) = −2.019, *p* = 0.017), higher MCP4 (t(94) = 1.966, *p* = 0.022) and higher TARC (t(94) = 2.282, *p* = 0.029). Subsequently, the medicated group had small reductions in IL-10 over time, while the levels of unmedicated participants increased (interaction F(1,48) = 5.193, *p* = 0.027); see Figure 3.

Log transformed mean IL-10 values over time (error bars show SEM); interaction with psychotropic medication use. For clarity of the Figure 3, a value of 1 has been added to protein data in the graph. Log values for unmedicated participants (*n* = 26): pre-treatment mean = −0.668, SEM = 0.053, post-treatment mean = −0.581, SEM = 0.055. Medicated participants (*n* = 24): pre-treatment mean = −0.643, SEM = 0.055, post-treatment mean = −0.664, SEM = 0.057.

#### 3.5.3. Associations between Inflammation and Treatment Resistance

More treatment-resistant participants had higher baseline levels of TARC (r = 0.514, *p* < 0.001), IL-12 (r = 0.353, *p* = 0.01), IL-6 (r = 0.278, *p* = 0.046) and lower levels of IFNy (r = 0.317, *p* = 0.022). Levels of TARC (r = 0.421, *p* = 0.002), IL-12 (r = 0.396, *p* = 0.004) and IFNy (r = 0.292, *p* = 0.040) at post-treatment were similarly associated with MSM score. 

## 4. Discussion 

These findings provide an insight into inflammatory changes that can occur alongside naturalistic psychological treatment, even in the absence of clinical recovery from depression. Results help to clarify some of the inflammatory associations with depression over time and with response to treatment, incorporating indications into the activity of a range of inflammatory markers across subtypes of depressed individuals undergoing psychological treatment. The therapy response rate of 49% is comparable to those previously reported for IAPT psychological therapy services [33]. Treatment non-responders had more severe depression and more psychiatric comorbidities before starting treatment, which generally aligns with previous literature [34,35].

### 4.1. Inflammatory Relationships with Treatment Response

Elevated pro-inflammatory cytokines TNFα and IL-6 were prospectively predictive of subsequent non-response to psychological therapy, which replicates previous findings for both these biomarkers in a multimodal inpatient treatment setting for patients with severe treatment-resistant depression [21], and in antidepressant trials [36,37]. Both of these cytokines are highly related functionally in their important involvement in early acute inflammation initiating expression of additional proteins, that include sICAM1 which has not been examined in previous studies of psychological treatments for depression [38] and was also found to predict non-response in this study. sICAM1 is a chemoattractant adhesion molecule and immunoglobulin which is involved with a variety of chemokines in assisting lymphocyte responses to inflammation, regulated by TNFα and IL-6 [39]. Our findings of increased inflammation (IL-6, CRP, MCP4, TARC) also align with previous research finding increased pro-inflammatory cytokines in treatment-resistant versus responsive patients with depression [40]. CRP in particular has previously been found to correlate with depression severity after treatment [41] and similar findings have been identified for MCP4 and TARC although the latter have scarcely been examined in depression intervention studies [21].

In the only RCT to date assessing inflammatory predictors to psychological therapies, Harley et al. analysed two studies whose methodology was almost identical: one antidepressant and one psychotherapy trial for MDD. Elevated CRP predicted a poor response to interpersonal therapy or CBT, but a good response to nortriptyline or fluoxetine [10]. The authors did not examine the longitudinal effects of these treatments on inflammatory activity, but their findings might indicate psychological or psychotropic treatment categorisation as a potential candidate on which to stratify interventions using inflammatory markers if sufficiently replicated.

In general, our results suggest a pro-inflammatory state in non-responders characterised by the presence of high levels of prominent Th1 cytokines TNFα and IL-6 as well as CRP signalling an early immune response, in combination with the related cell-adhesion molecule sICAM1 and chemokines MCP4 and TARC, which work together with cell adhesion molecules on the endothelial surface. It is worth noting that, in line with previous work, increased inflammation is found in participants who have experience of treatment-resistance within the current episode retrospectively (in this case, for TARC, IL-12 and IL-6) [21].

There is one protein whose findings appear at odds with the other results reported. The inverse findings of low IFNγ in more severely ill participants is consistent across a number of comparisons, with low IFNy predictive of subsequent therapy non-response, associated cross-sectionally with non-response, negatively correlated with depression severity and particularly the somatic symptoms as well as negative correlations with treatment-resistance severity (MSM score). These effects suggest some degree of Th1 inflammatory imbalance in these patients. IFNγ is principally produced by natural killer cells (as well as activated T-cells); decreased natural killer cell activity has been reported in depression previously, found not to alter with treatment [42] and it is possible that low natural killer cell activity could contribute to attenuated IFNγ leading to inflammatory system imbalances. This is supported by meta-analytic estimates of reduced IFNγ in those with MDD compared to non-affected controls [43].

### 4.2. Relationships between Inflammation and Depression Severity

Cross-sectional inflammatory correlations with PHQ scores firstly support previous evidence that inflammatory dysregulations are greater in those with more severe depression, with significant associations emerging for markers implicated in treatment response as discussed above (TNFa, sICAM1, IL-6, TARC, MCP4 and IFNy) as well as related proteins Mip1b, MCP1, SAA [17,18,21]. The key finding from these comparisons is the absence of correlations between inflammatory protein levels and the cognitive/psychological symptoms subscale scores, while significant correlations with the somatic subscale were widespread. 

### 4.3. Inflammatory Relationships between Symptom Subtypes

In light of the aforementioned inflammatory associations with somatic symptom severity, it is perhaps surprising that few proteins differed between participants with a prominently somatic versus cognitive subtype when grouped. IL-7 is tentatively higher in participants with a primarily somatic subtype at baseline, while IL-6 and SAA decrease over time in those with a somatic but not cognitive subtype of depression. Considering these findings as a whole, we may speculate that there is a stronger relationship between inflammation and somatic presentations than cognitive or emotional presentations of depression, which supports Duivis et al.’s [17] previous findings. This effect may be more pronounced than either the current study or previous studies have observed, due to the relatively crude measurement used to define a somatic subtype. Further investigation is warranted to establish whether the few significant findings identified here signify meaningful differences between patients with predominantly somatic or non-somatic symptoms. The severity of specific symptoms mirroring sickness behaviour (which are slightly distinct from those considered here) may provide another target for an inflammatory symptom subtype, as has been explored recently [18].

### 4.4. Inflammatory Relationships between Diagnostic Subtypes

Bipolar depression is associated with reductions in Tie2 and sVCAM1 over time while these remain high in unipolar participants. These markers have rarely been examined cross-sectionally [21] and not to our knowledge longitudinally, between these groups, but could represent subtly different mechanisms between these mood disorders in terms of inflammatory signalling or small differences driven by possible affective lability, or medication differences, although these were not detected using the method employed. True effects may also have been masked due to reduced sample size at post-treatment. Approximately one third of participants met criteria for a bipolar disorder, which accords with previous reports [44] although the measures used to assess bipolarity (MINI or HCL where missing) have been criticised for over-indicating bipolar disorders [45]. As well as the possibility of false-positive bipolar-grouped participants, this sample may not be representative of those with bipolar depression as many were either treated with a monoaminergic antidepressant monotherapy (i.e., without co-prescription of a mood stabiliser) or were unmedicated. It is likely that most patients had experienced only mild hypomanic episodes. Nevertheless, these are key areas to be addressed in future, hypothesis-driven research.

### 4.5. Longitudinal Changes between Pre- and Post- Therapy Timepoints

IL-8 and IL-15 show strongly significant increases alongside treatment (surviving adjustment for multiple comparisons), while IL-17 and IL-7 increase with nominal statistical significance (*p* < 0.05). Clinical factors (treatment-response, medication use or prominent somatic/cognitive subtype) did not appear to affect these occurrences significantly (in contrast to previous findings [8]) and it is possible that part of the psychological therapy process influences inflammatory activity, although we have not been able to examine whether these changes are a result of lifestyle changes, activity levels, or, e.g., improvements to psychosocial functioning not detected by the binary response variable. As a well characterised pro-inflammatory chemokine, a rise in IL-8 levels is somewhat in contrast with other findings of inflammatory normalisation with therapy [6,7] although not all reviews have identified anti-inflammatory effects of therapy on inflammation for those with depression [9].

Despite this, all four proteins act in complex ways that are not exclusively pro-inflammatory. IL-8, for example, reduces leukocyte binding to endothelial cells. [46] The release of IL-8 is aided by IL-17 in endothelial cells and by both IL-15 and IL-7 in monocytes [46], which highlights some degree of functional connectivity between these proteins, although they were not inter-correlated in this study. Increases in IL-15 and IL-7 might be attributed to T-cell growth, which may equate with increases in monoamine activity, even in individuals with minor reductions in depression severity. 

The anti-inflammatory cytokine IL-10 was the only protein whose changes over time differed between patients with and without psychotropic medication use, increasing only in participants not taking medication. Some antidepressants can suppress IL-10 levels in the short- [47] and long- [48] term and it is possible that the suppressive effects of monoamine reuptake inhibitors are masked by additional neurotransmission-enhancing effects of psychological therapy, potentially including melatonin as well as serotonin and norepinephrine.

### 4.6. Strengths and Limitations

Firstly, it must be emphasised that most results discussed here are based on nominal statistical significance with results *p* < 0.05 rarely surviving Simes adjustment for multiple tests. This was justified by the current study being exploratory with low statistical power and the possibility that Simes led to type II error in minimising the risk of type I error [31]. However, in light of this, the results described should be interpreted with caution.

This study is clearly limited by low statistical power arising from small participant numbers and particularly the reduced follow-up sample, in conjunction with the number of comparisons. A related, and key, limitation of this work is the inability to account for all potentially important factors affecting circulating inflammatory proteins, spanning lifestyle factors (such as smoking, food and exercise), collection methods (non-standardisation of time of collection, fasting, etc.), clinical characteristics (e.g., number of episodes, age of onset), psychosocial factors (e.g., health-related quality of life or disability more broadly, recent stressors) and treatment characteristics (such as type, intensity and duration of therapy). There are a multitude of factors that influence these proteins, many of which also differ between depressed and non-depressed populations and indeed potentially between treatment responsive and resistant participants [12]. The common confounding factors that were considered in this study were selected based on their importance and rationale from previous evidence. Despite this, it is worth noting that some factors known to be highly involved with inflammation such as BMI were correlated with few markers in this study. A related point worthy of consideration is that pro-inflammatory elevations may represent a non-specific state of ‘ill health’ or ‘unwellness’ rather than as biomarkers specifically of depressive illness.

Research exploring homogenous subtypes, particularly those relating to inflammatory biomarkers, are scarce. A potential ‘somatic’ symptom subtype is addressed in the present study. Measurement criteria used to determine subtype categorisation are unstandardised and may not accurately reflect those participants experiencing a somatic-type mood disorder. A measure of atypical depression would have enabled an assessment of this subtype [11]. 

The MINI interview was used to categorise participants as having bipolar or unipolar depression. This has been validated as a diagnostic assessment tool, but for 5 participants the HCL-16 was used instead, due to missing MINI data. The HCL is a patient-rated screening tool and may be over-sensitive to detecting a bipolar diathesis. As with the characterisation of bipolarity, described above, participants were included as depressed based on a standardised patient-report screening method (using PHQ9 scores) but were not required to have a formal MDD diagnosis. On one hand, diagnostic accuracy of MDD has previously been suggested as important when assessing inflammatory abnormalities in depression [49]; conversely, the PHQ9 score cutoffs used have been validated as feasible for detecting clinically significant symptoms and for administering longitudinally, having additionally outperformed other widely-used measures at discriminating between treatment-response [50].

Using this approach to participant inclusion, our sample may have been more representative of those treated naturalistically in UK psychological therapy services and therefore have greater clinical utility depending on the findings of future studies assessing predictive models of response to psychological therapies for depression. 

### 4.7. Future Recommendations

A need for psychoneuroimmunological research to shift focus towards translational applications is being increasingly reported [2]. While further investigation into the inflammation-response link in depression is warranted, it is proposed that another primary focus should be the identification of potential subtypes that may include an ‘inflammatory’ component in depression. Somatic subtyping (dependent on measurement consensus) may correspond with inflammatory markers. Further elucidation of factors such as bipolarity and other psychiatric (as well as non-psychiatric) comorbidities is important and should be considered in controlled studies. Evaluating more reliable measurements of inflammation may help to enhance the consistency of findings between studies; RNA markers are more stable than proteins and have shown comparable associations with treatment response/resistance [51,52,53] as well as normalisation longitudinally with successful treatment [53]. However, RNA assays are costly and there may be scope for developing an ‘inflammatory risk index’ incorporating relevant factors that are feasible for use in clinical practice (e.g., CRP in conjunction with other relevant clinical and psychosocial measures) to more reliably assess inflammatory status and change over time.

### 4.8. Clinical Implications

If individuals can be prospectively identified as at risk for non-response to usual treatments, enhanced care and monitoring could be provided to these patients, to increase the probability of achieving remission. Particularly high levels of inflammatory markers and/or somatic symptoms may indicate stratification to more appropriate treatments (for instance a short-term course of an anti-inflammatory medication alongside a ‘stepped-up’ psychotherapy programme). If an inflammatory subtype is conceptualised and its nature established, it also has the potential for more accurate diagnosis and individualised antidepressant selection.

## 5. Summary and Conclusions

This exploratory study aimed to improve understanding of the relationships between subgroups of people with depression and their outcome to psychological therapy with a large panel of inflammatory proteins, measured before and after treatment. This study has demonstrated elevated inflammatory biomarkers in people with depression who are non-responsive to treatment. A poor outcome following psychological therapy is predicted prospectively by elevated IL-6, sICAM1 and TNFα, and attenuated IFNγ and concurrently by elevated CRP, MCP4, TARC and attenuated IFNy. Pro-inflammatory indications are also associated with retrospectively-assessed treatment resistance. A range of pro-inflammatory cytokines (TNFa, IL-6,), chemokines (TARC, Mip1a, MCP1, MCP4) chemoattractant (sICAM1) and acute phase (SAA) proteins are associated with the somatic symptoms of depression, with IL-6 and SAA showing decreases alongside therapy only in participants with a predominantly somatic subtype. IL-7, IL-17, IL-8 and IL-15 show overall increases alongside therapy, while IL-10 increases only in unmedicated participants. People with bipolar depression may demonstrate greater reductions in Tie2 and sVCAM1 with therapy. 

These findings have the potential to improve treatment selection and outcomes for people with depression, if a predictive/stratified model for response is validated and sufficiently replicated, with further improvements likely if an ‘inflammatory subtype’ of depression is discovered. Finally, these findings accord with the growing consensus that TNFα, IL-6 and CRP are among the important biomarkers with translational potential in mood disorders, suggesting that this may be true in the psychological as well as pharmacological treatment of depression.

## Figures and Tables

**Table 1:** Participant characteristics.

	Full Sample *N* = 96	Responders *n* = 47	Non-Responders *n* = 49	*p*-Value *
Gender n female (%)	68 (72%)	36 (77%)	32 (65%)	
Age Mean age (range)	37.7 (18–77)	35.5 (19–71)	39.8 (18–77)	
BMI Mean BMI (range)	25.8 (16–47) a	25.2 (16–47)	26.1 (18–45)	
Symptom subtypen somatic (%)	26 (33%) b	12 (31%)	14 (36%)	
Bipolar status n bipolar spectrum (%)	30 (31%)	15 (32%)	15 (31%)	
Childhood trauma severity Mean CTQ score (range)	52.2 (28–114) c	52 (28–114)	52.5 (28–102)	
Physical illness Mean MCIRS score (range)	16.4 (13–28) d	16.3 (13–28)	16.8 (13–25)	
Psychiatric medication n medicated (%)	45 (47%)	18 (38%)	27 (55%)	
Treatment-resistance Mean MSM score (range)	5.82 (3–10)	5.82 (3.5–9)	5.82 (3–10)	
Pre-treatment depression severity Mean PHQ9 score (range)	18.4 (10–27)	17.4 (10–25)	19.4 (10–27)	0.022
Post-treatment depression severity Mean PHQ9 score (range)	10.9 (0–27)	5 (0–21)	16.5 (10–27)	<0.001
Number of therapy sessions Mean number (range)	11.9 (4–35) e	10.8 (4–32)	13.2 (4–35)	
Number of psychiatric comorbidities Mean number (range)	3.2 (0–7)	2.6 (0–7)	3.6 (0–7)	0.015

* Non-significant unless stated. ^a^ *n* = 93 (46 responders, 47 non-responders); ^b^ *n* = 78 (39 responders, 39 non-responders); ^c^ *n* = 91 (45 responders, 46 non-responders); ^d^ *n* = 89 (45 responders, 44 non-responders); ^e^ *n* = 95 (47 responders, 48 non-responders).

**Table 2:** Inflammatory associations with psychological therapy response.

	Pre-Treatment Comparison					Post-Treatment Comparison				
Protein	OR	95% CI Lower Upper		*p*	adj. *p*	OR	95% CI Lower Upper		*p*	adj. *p*
**TNFα**	0.007	−9.686	−1.006	**0.016**	0.138	0.064	−10.210	2.810	0.310	0.466
**IL-6**	0.149	−3.496	−0.453	**0.011**	0.138	0.062	−7.081	−0.682	**0.027**	0.157
**CRP**	0.531	−1.418	0.145	0.091	0.419	0.216	−3.308	−0.446	**0.006**	0.067
IL-10	0.629	−1.786	0.783	0.417	0.779	0.313	−3.415	1.191	0.239	0.466
IL-8*	0.393	−3.430	1.681	0.440	0.779	0.038	−10.259	0.846	0.148	0.369
IL-12	0.992	−1.940	1.773	0.991	0.991	1.974	−3.401	3.312	0.609	0.643
IL-7	1.080	−2.171	2.780	0.954	0.991	0.094	−7.587	1.901	0.257	0.466
IL-15	1.896	−3.022	4.221	0.728	0.887	0.016	−11.896	1.615	0.150	0.369
IL-16	0.871	−2.823	2.524	0.915	0.991	0.225	−6.226	3.171	0.504	0.592
IL-17	1.626	−0.799	1.811	0.409	0.779	1.232	−1.760	2.338	0.817	0.831
MCP1	0.611	−3.312	1.886	0.733	0.887	0.072	−10.644	1.082	0.285	0.466
**MCP4**	0.754	−2.467	1.757	0.783	0.901	0.016	−10.689	−0.680	**0.025**	0.157
Mip1b	0.188	−4.530	0.563	0.174	0.572	0.924	−4.747	4.327	0.962	0.962
Eotaxin	0.517	−2.944	1.393	0.485	0.783	0.432	−4.118	1.907	0.553	0.604
**sICAM1**	0.010	−9.293	−0.557	**0.018**	0.138	0.002	−14.894	−0.371	**0.037**	0.159
sVCAM1	0.278	−6.311	3.308	0.577	0.783	0.011	−15.061	2.040	0.247	0.466
SAA	0.639	−1.527	0.735	0.425	0.779	0.264	−4.406	0.067	0.112	0.323
TARC *	0.453	−2.512	0.733	0.304	0.779	0.030	−6.469	−1.723	**0.003**	0.067
Tie2	0.076	−6.965	−0.134	0.140	0.537	0.185	−8.260	1.213	0.370	0.466
IP-10	0.348	−4.120	0.870	0.382	0.779	0.153	−7.253	2.033	0.348	0.466
**IFNγ**	5.205	0.102	3.755	**0.042**	0.242	5.743	−0.338	4.334	0.079	0.271
Eotaxin3	0.781	−1.184	0.725	0.579	0.783	0.343	−4.240	0.590	0.290	0.466
TNFβ *	0.765	−1.255	0.576	0.553	0.783	0.621	−2.251	1.013	0.509	0.592

Results of unadjusted univariate logistic regressions of inflammation (IV) on responder vs. non-responder group (DV) after psychological treatment. Bold protein names and *p* values indicate significant effects. At pre-treatment the model r^2^ ranged from <0.001 (IL-12, IL-7, IL-16) to 0.090 (TNFa). After treatment the model r^2^ ranged from <0.001 (Mip1b) to 0.236 (TARC). OR = odds ratio, equivalent to standardised beta coefficient, 95% CI = 95% confidence intervals, *p* = unadjusted *p*, before Simes control for multiple testing, adj. *p* = *p* value after Simes control for multiple comparisons. * Hosmer–Lemeshow test *p* < 0.05, indicating possible poor model fitting (all at post-treatment only). When adjusting for age, gender and BMI each regression model fit was satisfactory. All other regression models were adequately fitted according to the Hosmer–Lemeshow test.

**Table 3:** Inflammatory changes over treatment period in responders and non-responders.

Protein	Before vs. after Treatment			Treatment x Response Interaction		
	*F*	*p*	adj. *p*	*F*	*p*	adj. *p*
TNFα	0.023	0.880	0.950	0.015	0.902	0.909
IL-6	0.004	0.950	0.950	0.032	0.859	0.909
CRP	0.022	0.883	0.950	0.068	0.795	0.909
IL-10	3.164	0.082	0.236	2.167	0.147	0.676
**IL-8**	14.600	**0.0009**	**0.021**	2.352	0.132	0.676
IL-12	0.842	0.363	0.816	0.542	0.465	0.901
**IL-7**	7.181	**0.010**	0.077	0.188	0.666	0.901
**IL-15**	9.826	**0.003**	**0.035**	0.345	0.560	0.901
IL-16	0.328	0.511	0.840	2.301	0.136	0.676
**IL-17**	5.049	**0.027**	0.167	3.058	0.087	0.676
MCP1	3.322	0.075	0.236	1.455	0.234	0.769
MCP4	0.110	0.742	0.948	0.074	0.787	0.909
Mip1b	0.557	0.459	0.816	0.013	0.909	0.909
Eotaxin	0.170	0.682	0.948	0.287	0.595	0.901
sICAM1	0.173	0.680	0.948	0.015	0.903	0.909
sVCAM1	2.850	0.098	0.250	2.628	0.112	0.676
SAA	0.688	0.411	0.816	0.349	0.558	0.901
TARC	0.553	0.461	0.816	0.333	0.567	0.901
Tie2	0.008	0.928	0.950	0.851	0.361	0.901
IP-10	0.076	0.784	0.949	0.224	0.638	0.901
IFNγ	3.440	0.070	0.236	0.301	0.586	0.901
Eotaxin3	0.142	0.708	0.948	0.019	0.892	0.909
TNFβ	3.403	0.071	0.236	1.532	0.222	0.769

Results of 2 × 2 mixed-factorial ANOVA comparing protein changes over time (pre- to post-treatment) between responders and non-responders. The before vs. after treatment columns describe the main effect of cytokine comparison between time points in the full sample and the Treatment x response interaction column describes the effect of response status on inflammatory change over time (n = 51). Bold protein names and *p* values indicate significant effects. F = ANOVA test statistic (degrees of freedom 1, 48), *p* = uncorrected *p*, before Simes control for multiple comparisons, adj. *p* = *p* value after Simes control for multiple comparisons.

## Supplementary Materials

The following are available online at https://www.mdpi.com/2077-0383/9/12/3918/s1, Supplementary S1: Table describing inflammatory marker levels (descriptive statistics) in therapy responders and non-responders. Supplementary S2: Text describing post-hoc analyses comparison inflammatory proteins with treatment response, adjusting for other potentially important confounders.
