# Supplementary material for: Inflammatory Proteins and Clinical Response to Psychological Therapy in Patients with Depression: An Exploratory Study
Source: J Clin Med. 2020 Dec 2;9(12):3918. doi: 10.3390/jcm9123918 (PMC7761611; doi:10.3390/jcm9123918)
Supplement: Supplementary file 1 [file jcm-09-03918-s001.pdf]

Journal of
*Clinical Medicine*

MDPI

**Supplement 1 Inflammatory marker levels in therapy responders and non-responders.**

| | ----- Pre-treatment comparison ----- | | | | ----- Post-treatment comparison ----- | | | |
|---|---|---|---|---|---|---|---|---|
| | Responder | | Non-responder | | Responder | | Non-responder | |
| Protein | lg-mean | SD | lg-mean | SD | lg-mean | SD | lg-mean | SD |
| TNFα | 0.289 | 0.108 | 0.348 | 0.303 | 0.108 | 0.338 | 0.123 | 0.111 |
| IL-6 | -0.318 | 0.286 | -0.170 | -0.298 | 0.298 | -0.079 | 0.299 | 0.276 |
| CRP | 6.201 | 0.539 | 6.388 | 6.172 | 0.506 | 6.599 | 0.568 | 0.557 |
| IL-10 | -0.732 | 0.407 | -0.675 | -0.652 | 0.289 | -0.567 | 0.259 | 0.304 |
| IL-8 | 0.951 | 0.182 | 0.979 | 1.012 | 0.130 | 1.081 | 0.175 | 0.165 |
| IL-12 | 1.307 | 0.163 | 1.305 | 1.359 | 0.144 | 1.415 | 0.180 | 0.181 |
| IL-7 | 1.967 | 0.222 | 1.968 | 1.970 | 0.200 | 1.937 | 0.264 | 0.278 |
| IL-15 | 0.447 | 0.105 | 0.439 | 0.481 | 0.115 | 0.530 | 0.105 | 0.127 |
| IL-16 | 2.325 | 0.177 | 2.329 | 2.300 | 0.147 | 2.334 | 0.163 | 0.162 |
| IL-17 | 0.256 | 0.390 | 0.199 | 0.309 | 0.347 | 0.288 | 0.279 | 0.303 |
| MCP1 | 2.457 | 0.152 | 2.470 | 2.467 | 0.168 | 2.549 | 0.218 | 0.172 |
| MCP4 | 2.170 | 0.189 | 2.182 | 2.142 | 0.151 | 2.272 | 0.220 | 0.226 |
| Mip1b | 2.089 | 0.193 | 2.145 | 2.115 | 0.159 | 2.117 | 0.135 | 0.179 |
| Eotaxin | 2.296 | 0.208 | 2.326 | 2.317 | 0.213 | 2.359 | 0.254 | 0.223 |
| sICAM1 | 6.481 | 0.415 | 6.546 | 6.413 | 0.468 | 6.646 | 0.332 | 0.353 |
| sVCAM 1 | 5.668 | 0.097 | 5.717 | 5.684 | 0.100 | 5.758 | 0.124 | 0.111 |
| SAA | 5.775 | 0.097 | 5.785 | 5.782 | 0.092 | 5.825 | 0.114 | 0.085 |
| TARC | 2.472 | 0.226 | 2.526 | 2.451 | 0.266 | 2.720 | 0.336 | 0.297 |
| Tie2 | 3.625 | 0.192 | 3.703 | 3.607 | 0.223 | 3.682 | 0.231 | 0.176 |
| IP-10 | 2.326 | 0.154 | 2.368 | 2.332 | 0.151 | 2.383 | 0.191 | 0.241 |
| IFNγ | 0.707 | 0.285 | 0.592 | 0.701 | 0.316 | 0.538 | 0.320 | 0.249 |
| Eotaxin 3 | 0.742 | 0.491 | 0.795 | 0.743 | 0.496 | 0.916 | 0.404 | 0.442 |
| TNFβ | -0.725 | 0.541 | -0.663 | -0.664 | 0.469 | -0.578 | 0.388 | 0.428 |

Protein levels before and after therapy in responders and non-responders.

lg-mean = log-transformed mean value in pg/ml (except for CRP which was converted into mg/L), SD = standard deviation.

**Supplement 2: Post-hoc analyses adjusting for other potentially important confounders**

Elevated IL-6 at baseline predicted non-response. Baseline IL-6 was also associated with age and physical illness, so a post-hoc logistic regression including these two variables was conducted. In the absence of indicated multi-collinearity, IL-6 (and neither other variable) remained significant: OR = 0.153 [95% CI 0.027, 0.861], p = 0.033.

Elevated TNFa at baseline predicted non-response. Baseline TNFa was also associated with age, physical illness and the number of comorbidities. A post-hoc logistic regression including these three variables indicated that, without significant multi-collinearity, TNFα remained a significant predictor of non-response (OR = 0.009 [95% CI 0.000, 0.698], p = 0.034 while none of the other variables appeared significantly associated.

Attenuated IFNy at baseline predicted non-response. Baseline IFNy was also negatively associated with early life stress (CTQ score). In the post-hoc logistic regression containing these two variables, IFNy was only associated with response at a trend level (OR = 5.178 [95% CI 0.974, 27.536], p = 0.054) while CTQ was not a significant predictor.

After treatment, non-response was cross-sectionally associated with elevated CRP; CRP was positively associated with age (already adjusted for in a priori logistic regression) and therefore a subsequent regression was not conducted.

After treatment, non-response was cross-sectionally associated with elevated MCP4, which was also correlated with age and physical illness. A post-hoc logistic regression including these factors suggested that only MCP4 was independently associated with non-response (OR = 0.020 [95% CI 0.000, 0.942], p = 0.047).

After treatment, non-response was cross-sectionally associated with elevated TARC. TARC was also positively associated with physical illness. A post-hoc logistic regression including these two variables indicated that TARC (but not physical illness) remained associated with non-response (OR = 0.030 [95% CI 0.002, 0.520], p = 0.016) although poor model fitting was indicated (Hosmer and Lemeshow Chi-square statistic 17.359, p = 0.027).

After treatment, non-response was cross-sectionally associated with attenuated IFNy. At this timepoint, IFNy was also negatively associated with number of psychiatric comorbidities. The post-hoc logistic regression including these two variables rendered IFNy a non-significant contributor (OR = 2.716 [95% CI 0.283, 26.075], p = 0.387) whereas the number of comorbidities was higher in non-responders (OR = 0.640 [95% CI 0.423, 0.969], p = 0.035).

When not adjusting for age, gender and BMI, IL-6 was significantly higher in non-responders. IL-6 was indeed positively associated with age, but in the post-hoc logistic regression containing only IL-6 and age, IL-6 remained weakly significant (OR = 0.062 [95% CI 0.004, 0.924], p = 0.044) while age was not significantly associated with response.

Similarly, when not adjusting for age, gender and BMI, sICAM1 was significantly higher in non-responders. sICAM was also associated with age as well as physical illness. The post-hoc logistic regression including these three factors suggested that none of the variables were significantly and independently associated with response (sICAM1 being the strongest contributor with OR = 0.002 [95% CI 0.000, 1.540), p = 0.066).
